# Genome-wide association analyses reveal significant loci and strong candidate genes for growth and fatness traits in two pig populations

**DOI:** 10.1186/s12711-015-0089-5

**Published:** 2015-03-14

**Authors:** Ruimin Qiao, Jun Gao, Zhiyan Zhang, Lin Li, Xianhua Xie, Yin Fan, Leilei Cui, Junwu Ma, Huashui Ai, Jun Ren, Lusheng Huang

**Affiliations:** Key Laboratory for Animal Biotechnology of Jiangxi Province and the Ministry of Agriculture of China, Jiangxi Agricultural University, Nanchang, China

## Abstract

**Background:**

Recently, genome-wide association studies (GWAS) have been reported on various pig traits. We performed a GWAS to analyze 22 traits related to growth and fatness on two pig populations: a White Duroc × Erhualian F_2_ intercross population and a Chinese Sutai half-sib population.

**Results:**

We identified 14 and 39 loci that displayed significant associations with growth and fatness traits at the genome-wide level and chromosome-wide level, respectively. The strongest association was between a 750 kb region on SSC7 (SSC for *Sus scrofa*) and backfat thickness at the first rib. This region had pleiotropic effects on both fatness and growth traits in F_2_ animals and contained a promising candidate gene *HMGA1* (*high mobility group AT-hook 1*). Unexpectedly, population genetic analysis revealed that the allele at this locus that reduces fatness and increases growth is derived from Chinese indigenous pigs and segregates in multiple Chinese breeds. The second strongest association was between the region around 82.85 Mb on SSC4 and average backfat thickness. *PLAG1* (*pleiomorphic adenoma gene 1*), a gene under strong selection in European domestic pigs, is proximal to the top SNP and stands out as a strong candidate gene. On SSC2, a locus that significantly affects fatness traits mapped to the region around the *IGF2 (insulin-like growth factor 2*) gene but its non-imprinting inheritance excluded *IGF2* as a candidate gene. A significant locus was also detected within a recombination cold spot that spans more than 30 Mb on SSCX, which hampered the identification of plausible candidate genes. Notably, no genome-wide significant locus was shared by the two experimental populations; different loci were observed that had both constant and time-specific effects on growth traits at different stages, which illustrates the complex genetic architecture of these traits.

**Conclusions:**

We confirm several previously reported QTL and provide a list of novel loci for porcine growth and fatness traits in two experimental populations with Chinese Taihu and Western pigs as common founders. We showed that distinct loci exist for these traits in the two populations and identified *HMGA1* and *PLAG1* as strong candidate genes on SSC7 and SSC4, respectively.

**Electronic supplementary material:**

The online version of this article (doi:10.1186/s12711-015-0089-5) contains supplementary material, which is available to authorized users.

## Background

Domestic pigs display great phenotypic diversity, that is attributable to approximately 10 000 years of natural and artificial selection [1]. Currently, Western commercial pigs show divergent phenotypes compared to Chinese indigenous pigs. Western commercial breeds, such as Large White, Landrace, Duroc and Pietrain, have experienced intensive selection for lean pork production in the past decades. Their excellent performance, including fast growth and a high lean percentage, has led them to dominate the global pig industry. Conversely, Chinese indigenous breeds have been historically selected for fat deposition, since fat was an important source of energy for Chinese farmers in ancient times. These breeds are characterized by obesity and a slow growth rate but good meat quality and excellent adaptability to diverse environments. In general, Chinese native pigs have average daily gains of ~400 g/d and a lean percentage of less than 45%, which are very different from the average daily gain of more than 800 g/d and a lean percentage greater than 60% in Western commercial pigs [2].

To dissect the molecular basis of the divergent phenotypes seen between Chinese and Western pigs, researchers have established multiple F_2_ intercross populations using Chinese and Western breeds as founder animals [3-6]. Genome scans have been performed on these experimental populations using sparse microsatellite markers across the pig genome to identify quantitative trait loci (QTL) for a variety of traits. For growth and fatness traits, a total of 2623 QTL have been deposited to date in the pig QTL database (http://www.animalgenome.org/cgi-bin/QTLdb/SS/index). Notably, QTL that are significantly associated with growth and fatness have been consistently identified on pig chromosomes (SSC for *Sus scrofa* chromosome) 1, 2, 4, 6, 7 and X using F_2_ intercross populations of Chinese and Western origin [3-6]. These findings have advanced our understanding of the genetic architecture of porcine growth and fatness traits. Nevertheless, the resolution of traditional QTL mapping is relatively poor due to the limited number of recombination events in the F_2_ crosses; confidence intervals are generally on the order of ~20 cM. Such large regions can contain an abundance of genes, which hampers the prioritization of plausible candidate genes. Thus, the causal variants that underlie the identified QTL remain poorly understood. To date, only one nucleotide, in intron 3 of the *IGF2 (insulin-like growth factor 2*) gene on SSC2, has been convincingly shown to cause a major QTL effect on muscle mass and heart weight [7].

Recently, genomic tools have revolutionized genetic studies of complex traits. Large human or animal populations can be affordably genotyped using high-density SNP (single nucleotide polymorphism) arrays. The resulting data, together with advanced statistical tools, allow researchers to perform genome-wide mapping of loci for phenotypic traits with greater power and higher accuracy than with traditional QTL mapping. The so-called genome-wide association study (GWAS) mapping approach has been successfully implemented in a growing list of species, including pigs [8-12]. Here, we report the results of GWAS mapping of loci for porcine growth and fatness traits using two experimental populations: a White Duroc × Erhualian F_2_/F_3_ intercross population and a Chinese Sutai half-sib population. The findings confirm our previous QTL mapping results and, more importantly, reveal a list of novel loci, refine the confidence intervals, and identify strong candidate genes for the major and multifaceted QTL on SSC4 and SSC7.

## Methods

### Ethics statement

All procedures involving animals were in compliance with the guidelines for the care and use of experimental animals established by the Ministry of Agriculture of China. The ethics committee of Jiangxi Agricultural University specifically approved this study.

### Animals and phenotypes

Two experimental populations were used in this study: a White Duroc × Erhualian F_2_/F_3_ intercross population and a Chinese Sutai half-sib population. The White Duroc × Erhualian intercross population was established as previously described [13]. Briefly, two White Duroc boars were mated to 17 Erhualian sows. From this first cross, nine F_1_ boars and 59 F_1_ sows were then intercrossed to produce a total of 1912 F_2_ animals in six batches and 560 F_3_ animals in two batches. Sutai pigs are a Chinese synthetic breed that is derived from Chinese Taihu (50%) and Western Duroc (50%) following more than 18 generations of artificial selection. All piglets were weaned at day 46 and males were castrated at day 90. All fattening pigs were raised under controlled indoor conditions in the experimental farm of Jiangxi Agricultural University (China) and were fed ad libitum on a diet containing 16% crude protein, 3100 kJ digestible energy and 0.78% lysine. Pigs were slaughtered for phenotype recording at the age of 240 ± 3 days. Body weight was measured at birth and at days 21, 46, 120, 210 and 240 for each F_2_ and F_3_ individual. For Sutai pigs, body weight was also recorded at these growth stages except at day 46. Average daily weight gain at multiple time intervals was calculated. After slaughter, seven fatness-related traits, including backfat thickness at the shoulder, the first rib, the last rib and the hip, and weight of leaf fat, veil fat and abdominal fat, were recorded for all individuals from both populations. A total of 930 F_2_ individuals and 432 Sutai pigs from five sires and 60 dams were used for GWAS mapping in this study.

### Genotypes and quality control

Genomic DNA of each animal was extracted from ear or tail tissue using a standard phenol/chloroform method. A total of 1017 animals, including 930 F_2_ pigs, 87 parental pigs in the F_2_ cross and 432 Sutai pigs, were genotyped for 62 163 SNPs using the Illumina PorcineSNP60 BeadChip according to the manufacturer’s protocol. Quality control procedures were carried out using Plink v 1.07 [14], and the same quality control criteria were applied to the SNP data from the two populations. Briefly, animals with a call rate greater than 0.9 and mendelian error rate smaller than 0.05, and SNPs with a call rate greater than 0.9, minor allele frequency higher than 0.05, *P* value greater than 10^−6^ for the Hardy-Weinberg equilibrium test and mendelian error rate smaller than 0.1 were included. A final set of 39 788 informative SNPs from 1017 animals from the F_2_ cross and the 432 Sutai pigs were used for subsequent analyses.

### Single-marker GWAS

The allelic effect of each SNP on phenotypic traits was tested using a general linear mixed model [15-17]. The model included a random polygenic effect, and the variance-covariance matrix was proportional to genome-wide identity by state [18]. The formula of the model was **Y** = **Xb** + **S**a + **Zu** + **e**, where **Y** is the vector of phenotypes; **b** is the estimator of fixed effects including sex, batch and carcass weight; a is the SNP substitution effect; and **u** is the vector of random additive genetic effects following the multinormal distribution **u** ~ N(0, **G**σ_α_^2^), in which **G** is the genomic relationship matrix that was constructed based on SNPs, as described in [19], and σ_α_^2^ is the polygenetic additive variance. **X** and **Z** are the incidence matrices for **b** and **u**, **S** is the incidence vector for a, and **e** is a vector of residual errors with a distribution of N(0, **I**σ_e_^2^). All single-marker GWAS analyses were conducted using the GenABEL package [20,21]. Based on the Bonferroni method, the genome-wide significance threshold was defined as 0.05/N, where N is the number of informative SNPs. The chromosome-wide significance threshold was defined as 0.1/N.

### Linkage disequilibrium and linkage association (LDLA) analysis

First, the haplotype of each chromosome was reconstructed for each animal in the F_2_ population using the 60 K SNP data, pedigree information and a Hidden Markov model [22]. The model simultaneously phased SNP genotypes and assigned the ensuing haplotypes to a predetermined number of ancestral haplotypes that was set at 20 in this study. Then, for each locus, the effect of these ancestral haplotypes was estimated using a mixed model framework: **Y** = **Xb** + **Zu** + **e** [23], where **Y** is the vector of phenotypes; **b** is the estimator of fixed effects including sex, batch and carcass weight; **X** and **Z** are the incidence matrices for **b** and **u**; **u** is the random additive genetic effect following the multinormal distribution **u** ~ N(0, **G**σ_α_^2^), in which **G** is the individual-individual similarities matrix which was calculated from whole-genome information and σ_α_^2^ is the polygenetic additive variance; and **e** is a vector of residual errors with a distribution of N(0, **I**σ_e_^2^). The ancestral haplotype-based LDLA analysis can use both within-family linkage information and across-family linkage disequilibrium information resulting from historical recombination events in ancestors of founder animals in the F_2_ population. We conducted the LDLA analysis using R scripts that were written for this purpose. The 95% confidence interval (CI) was determined by a LOD score drop-off of 2 from the value of the most significant loci.

### Haplotype analysis at the major loci on SSC7

For the major locus on SSC7, we constructed haplotypes for all 17 founder sows and two founder boars using the DualPHASE software [24]. The effects of the reconstructed haplotypes on phenotypic traits were evaluated using marker-assisted segregation analysis and multiple comparison tests, as described previously [7]. The putative critical region on SSC7 was identified by haplotype-sharing and LDLA-mapping analyses. Furthermore, haplotypes that corresponded to the refined critical region at the SSC7 locus were reconstructed for 589 individuals from 31 Chinese and Western breeds using 60 K SNPs from our previous study [25] and fastPHASE software [26]. Haplotypes with frequencies greater than 0.05 were used to construct a neighbor-joining tree in MEGA 5.0 with 1000 bootstrap iterations [27].

## Results

### Phenotypic values

Table S1 (see Additional file 1: Table S1) presents the phenotypic values of 14 growth traits and eight fatness traits measured in the White Duroc × Erhualian F_2_ population and Chinese Sutai pigs. Both populations showed comparable body weights at early stages, while the Sutai pigs had obviously lower body weights at adult ages (days 210 and 240). For fatness traits, Sutai pigs exhibited leaner phenotypes than the F_2_ animals; indeed, all fatness-related measurements were greater (*P* < 0.05) in F_2_ animals.

### Summary of GWAS results

In total, we identified 14 loci on seven chromosomes that exceeded the genome-wide significance thresholds; these included two loci on SSC2 and two on SSC3, four on SSC4, three on SSC7 and one each on SSC10, 14 and X. These prominent loci were associated with three growth traits and eight fatness traits (Table 1). However, none of them was shared between the two experimental populations. In addition, we detected 39 loci that demonstrated significance at the chromosome significant level (referred to as suggestive loci); these loci were located on all autosomes except SSC13, 16 and 17 (see Additional file 2: Table S2). Again, the F_2_ and Sutai populations showed distinct association signals at most of these loci. In Sutai pigs, no locus displayed a significant association to any fatness trait at the whole-genome level (see Additional file 3: Figure S1).

**Table 1:** **QTL of genome-wide significance identified by GWAS for growth and fatness traits in White Duroc × Erhualian F**_**2**_ **pigs and Sutai pigs**

**Chr** ^**1**^	**Trait**	**Abbreviation**	**Pop** ^**2**^	**N**_**snp**_^**3**^	**Top SNP**	**Position (bp)**	**MAF** ^**4**^	**Frequency** ^**5**^		**Effect**	***P*** **-value**	**Candidate gene**
								**Duroc**	**Erhualian**			
2	Body weight at day 21	BW21	Sutai	1	ss107909052	101694375	0.05	NA	NA	0.67	2.66E-08	*ARRDC3*
	Backfat thickness at the hip	HBFT	F_2_	4	ss131060419	920370	0.28	0.50	1.00	−0.25	2.59E-07	
3	Body weight at day 21	BW21	Sutai	4	ss131231938	130852806	0.06	NA	NA	1.05	6.70E-08	
	Average daily gain from day 0 to 21	ADG0-21	Sutai	2	ss131231938	130852806	0.06	NA	NA	0.06	1.22E-08	
4	Average backfat thickness	ABFT	F_2_	8	ss131269801	82850635	0.46	1.00	0.00	−0.28	8.50E-09	*PLAG1*
	Backfat thickness at the first rib	FRBFT	F_2_	4	ss131269801	82850635	0.46	1.00	0.00	−0.31	6.04E-08	
	Leaf fat weight	LFW	F_2_	6	ss131269801	82850635	0.46	1.00	0.00	−0.31	3.80E-08	
	Backfat thickness at the hip	HBFT	F_2_	4	ss131270942	88978451	0.44	1.00	0.00	−0.32	1.46E-08	
	Body weight at day 21	BW21	Sutai	4	ss131247062	134935929	0.05	NA	NA	1.09	1.35E-07	
	Average daily gain from day 0 to 21	ADG0-21	Sutai	2	ss131247062	134935929	0.05	NA	NA	0.06	1.48E-08	
	Average daily gain from day 0 to 21	ADG0-21	Sutai	2	ss107817770	133079451	0.08	NA	NA	0.06	1.48E-08	
7	Average backfat thickness	ABFT	F_2_	167	ss107837325	34803564	0.46	0.00	0.94	−0.62	7.63E-32	*HMGA1*
	Backfat thickness at the first rib	FRBFT	F_2_	184	ss107837325	34803564	0.46	0.00	0.94	−0.7	3.39E-32	
	Leaf fat weight	LFW	F_2_	165	ss107837325	34803564	0.46	0.00	0.94	−0.66	2.61E-30	
	Backfat thickness at the hip	HBFT	F_2_	155	ss107837325	34803564	0.46	0.00	0.94	−0.78	6.61E-28	
	Backfat thickness at the last rib	LRBFT	F_2_	166	ss107837325	34803564	0.46	0.00	0.94	−0.57	2.17E-24	
	Backfat thickness at the shoulder	SBFT	F_2_	115	ss107837325	34803564	0.46	0.00	0.94	−0.49	1.01E-17	
	Abdominal fat weight	AFW	F_2_	121	ss107837325	34803564	0.46	0.00	0.94	−0.15	1.79E-14	
	Veil fat weight	VFW	F_2_	29	ss107806758	35177641	0.45	0.00	0.88	−0.14	4.92E-08	
	Body weight at day 240	BW210	F_2_	3	ss131342496	32957768	0.49	0.00	1.00	−5.44	6.77E-07	
10	Average daily gain from day 0 to 21	ADG0-21	Sutai	1	ss478939281	13838740	0.08	NA	NA	0.02	8.89E-07	
14	Average daily gain from day 0 to 21	ADG0-21	Sutai	1	ss131505908	151638018	0.09	NA	NA	0.05	4.46E-08	
X	Backfat thickness at the hip	HBFT	F_2_	12	ss131561996	62086511	0.21	0.50	0.00	−0.31	6.84E-12	

^1^Chromosome; ^2^population; ^3^number of SNPs that surpass the genome-wide significance level; ^4^minor allele frequency; ^5^allele frequencies of top SNPs in two Duroc founder boars and 17 Erhualian founder sows from the F_2_ intercross resource population; NA, not available for Sutai pigs.

### Genome-wide significant loci and strong candidate genes in the F_2_ population

#### SSC7

**Figure 1:**
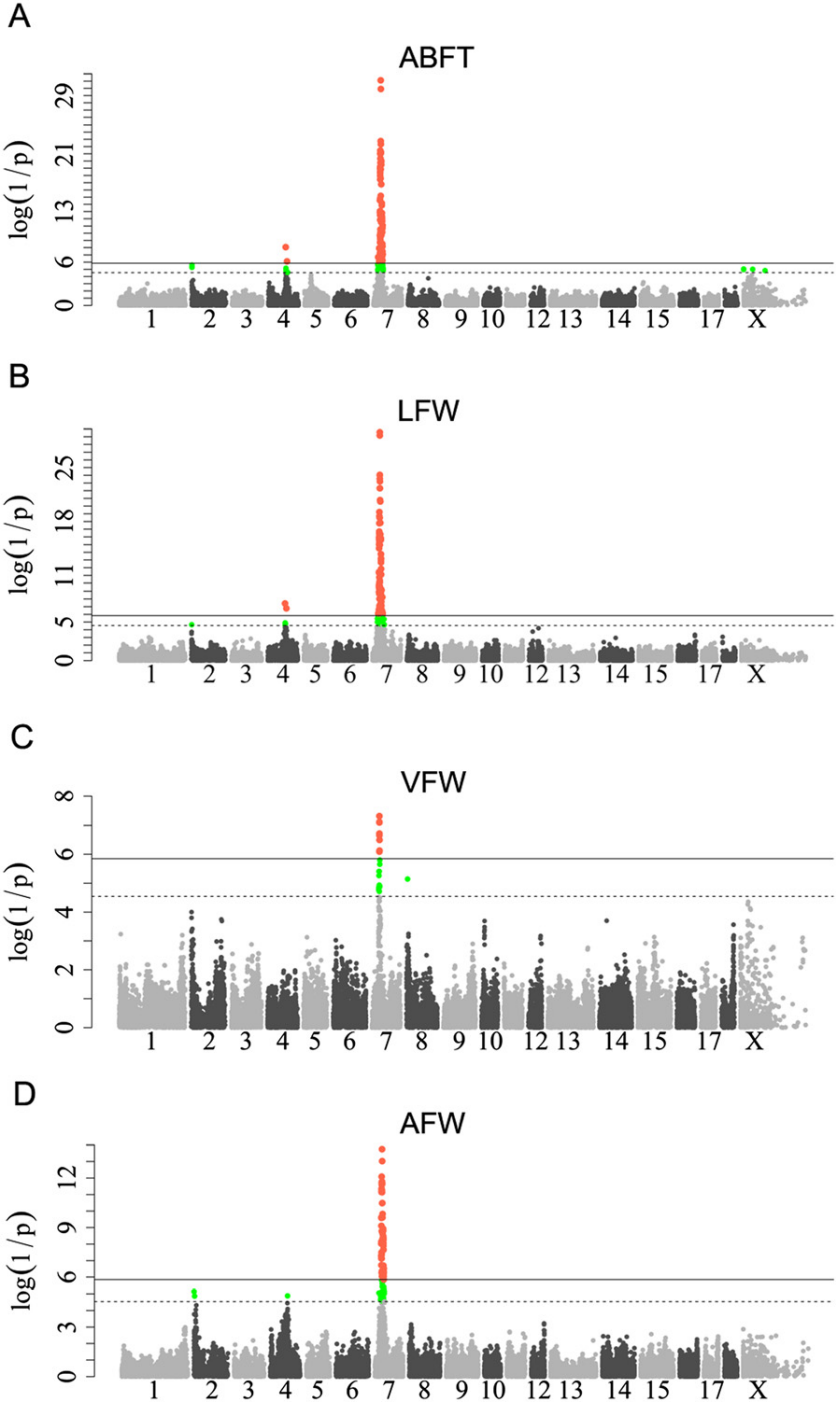
**Manhattan plots for the analyses of fatness traits in F**_**2**_ **animals**. GWAS results are shown for ABFT **(A)**, LFW **(B)**, VFW **(C)** and AFW **(D)** in F_2_ animals. In the Manhattan plots, negative log_10_ *P* values of the filtered high-quality SNPs were plotted against their genomic positions; SNPs on different chromosomes are denoted by different colors; solid and dashed lines indicate the 5% genome-wide and chromosome-wide (i.e., suggestive) Bonferroni-corrected thresholds, respectively; ABFT: average backfat thickness; LFW: leaf fat weight; VFW: veil fat weight; AFW: abdominal fat weight.

**Figure 2:**
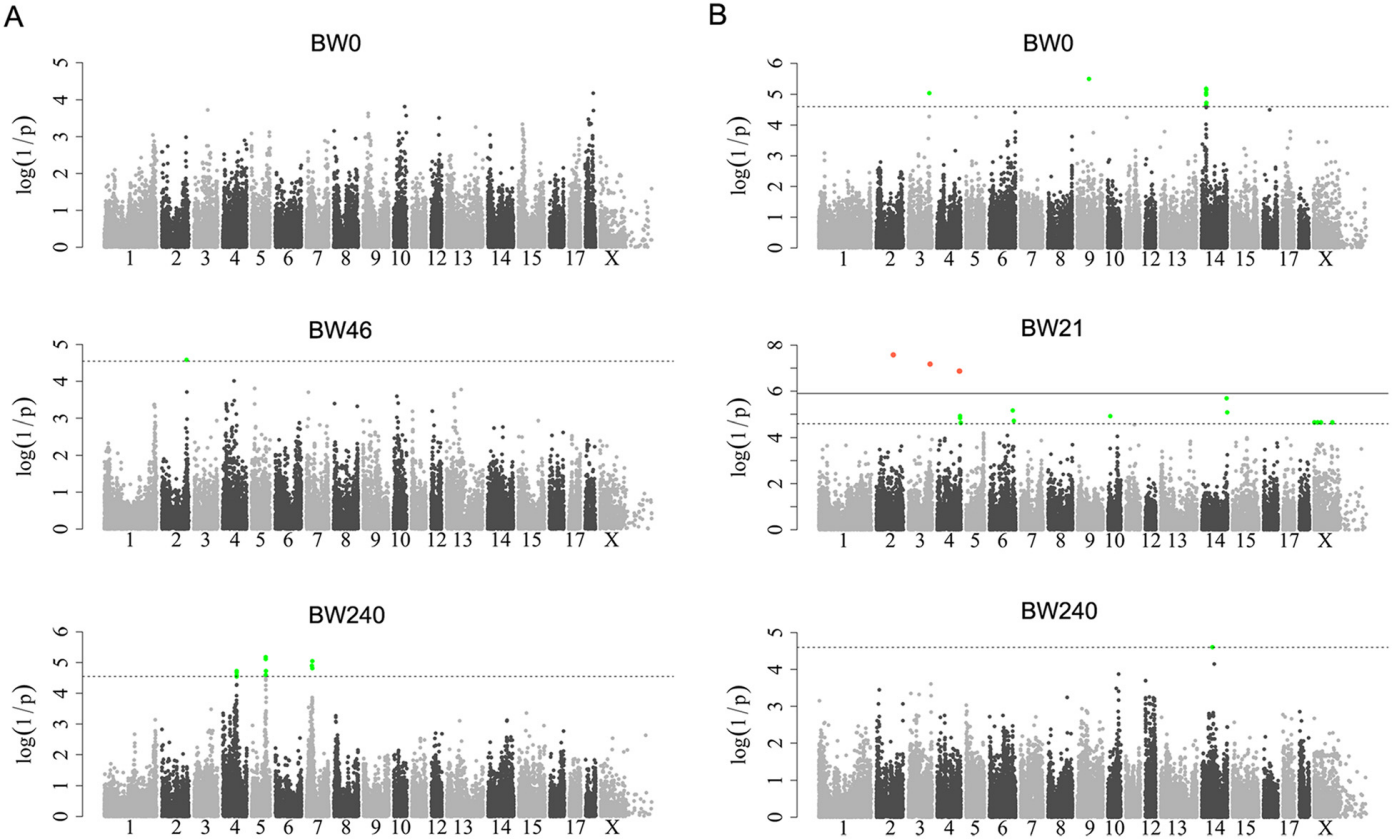
**Manhattan plots for the analyses of growth traits in F**_**2**_ **and Sutai pigs. (A)**: GWAS for birth weight (BW0), body weight at day 46 (BW46) and body weight at day 240 (BW240) in F_2_ animals. **(B)**: GWAS for BW0, BW21 and BW240 in Sutai pigs. In the Manhattan plots, negative log_10_ *P*-values of the filtered high-quality SNPs SNPs were plotted against their genomic positions; solid and dashed lines indicate the 5% genome-wide and chromosome-wide (i.e., suggestive) Bonferroni-corrected thresholds, respectively.

**Figure 3:**
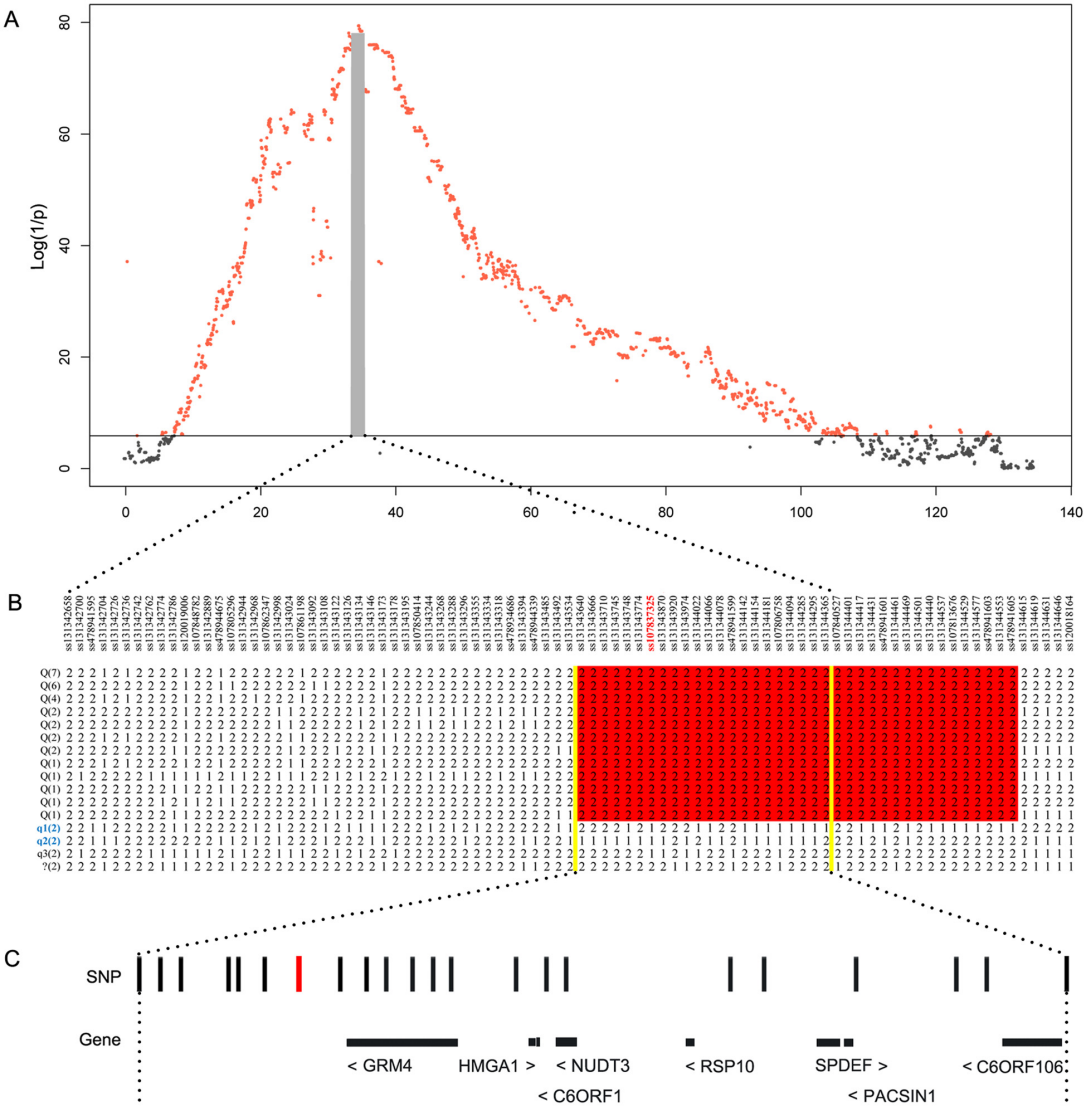
**Fine mapping of the major QTL for growth and fatness traits on SSC7 in the F**_**2**_ **population. (A)** Linkage disequilibrium and linkage analysis for average backfat thickness. In the Manhattan plots, negative log_10_ *P* values of the filtered high-quality SNPs SNPs are plotted against their genomic positions on SSC7; SNPs surpassing the 5% genome-wide significance threshold are denoted in red; solid line indicates the 5% genome-wide Bonferroni-corrected threshold; the 95% confidence interval (CI) defined by the LOD-dropoff-2 method is indicated by a gray shaded block. **(B)** Haplotype analysis for a 3.2-Mb region encompassing the 95% CI of the SSC7 locus in 17 F_0_ sows and 2 F_0_ boars. The allele with the higher frequency is denoted as 2, and the allele with the lower frequency is denoted as 1; the number of each haplotype in the 19 founder animals is given in the bracket; a total of 30 Q-bearing chromosomes corresponding to 12 haplotypes were derived from Erhualian founder sows, which shared a ~1.7-Mb segment, from ss131343640 to ss478941605; the shared 1.7-Mb segment containing the top SNP (ss107837325) is highlighted in red; six q-bearing chromosomes corresponding to three haplotypes were derived from two White Duroc F_0_ boars (individuals 73 and 75, q1 and q2) and two Erhualian F_0_ sows (individuals 124 and 126, q3). One haplotype, originating from Erhualian F_0_ individuals 142 and 146, is indicated by the symbol “?”; this haplotype is most likely a q-type haplotype; the ~750-kb region of overlap between the 95% CI and the Q-chromosome-containing segment is indicated by two vertical yellow lines. **(C)** The 22 informative SNPs and eight annotated genes in the 750-kb critical region. The top SNP (ss107837325) is marked in red.

Four major loci that affect fat deposition and growth were identified on SSC2, 4, 7 and X in the F_2_ population, which confirmed our previously published QTL mapping results [28]. Of these, the most striking locus was located at about 34 to 36 Mb on SSC7 (referred to hereafter as the SSC7 locus; Table 1) and was significantly associated with all fatness traits (Figure 1) and body weight at day 240 (Figure 2). The top SNP for the associated traits was ss107837325 at 34 803 564 bp on this chromosome. At this location, the allele substitution effect accounted for more than 5 mm of backfat thickness and 5.44 kg of body weight at day 240 (Table 1), explaining ~ 40% of phenotypic variance in these traits. By applying the LDLA and LOD-drop-off-2 approaches, we defined the 95% CI of the SSC7 locus as a 2.1-Mb region that was flanked by ss131342658 (33 299 125 bp) and ss131344365 (35 422 882 bp) (Figure 3A). Next, we conducted a haplotype analysis of a ~3.2-Mb region (between 33 299 125 and 36 475 968 bp) surrounding the 95% CI of the SSC7 locus, using 17 founder sows and two founder boars from the F_2_ population. We showed that a ~1.7-Mb segment between 34 673 190 (ss131343640) and 36 329 680 bp (ss478941605) was shared by 30 Erhualian founder chromosomes (Figure 3B). We then reconstructed haplotypes corresponding to this 1.7-Mb segment in all F_2_ animals and evaluated the effects of these haplotypes on average backfat thickness (ABFT) and leaf fat weight (LFW) in the F_2_ animals. The shared haplotype appeared to be a haplotype that was significantly associated with decreased fat deposition (referred to hereafter as Q haplotype) (see Additional file 4: Figure S2). This result was in accordance with previous reports [3,4,29-31], but was also unexpected considering that the Erhualian is an obese Chinese pig breed.

We noted that the haplotypes that were associated with increased fat deposition (referred to hereafter as q haplotypes) were derived from both White Duroc founder boars (q1 and q2, Figure 3B) and Erhualian founder sows (q3, Figure 3B). One haplotype (referred to as q?), originating from Erhualian founders 142 and 146, seemed to be a q-type haplotype (Figure 3B). However, the QTL status of this haplotype could not be deduced, since it was carried by only 10 of the 930 F_2_ animals that we had genotyped using the 60 K SNP panel (see Additional file 4: Figure S2A). To determine if this haplotype was a Q or a q haplotype, we further examined the White Duroc × Erhualian intercross population. In our previous study [32], we had genotyped all F_2_ and F_3_ descendants of individuals 142 and 146, for a total of 20 markers around the SSC7 QTL region. When we examined this marker and pedigree information, we found one additional F_2_ animal (2045) and six F_3_ animals that had inherited the q? haplotype from individuals 142 and 146 (see Additional file 5: Figure S3). We then conducted further statistical analyses on the phenotypic data of the 17 animals. We found that individuals carrying the q? haplotype had obviously higher ABFT and LFW than those with the Q haplotype (see Additional file 4: Figure S2B). We thus assumed that the q? haplotype is a q-type haplotype. To obtain further evidence for this assumption, we compared the phenotypic differences in ABFT and LFW between q?, q-, and Q-type haplotype pairs (see Additional file 6: Table S3). The pair-wise *t*-test statistics indicated that there was no significant difference in ABFT (*P* = 0.10) and LFW (*P* = 0.28) between qq and qq? individuals. Additionally, there was no significant difference in ABFT (*P* = 0.29) and LFW (*P* = 0.29) between Qq and Qq? individuals. In comparison, QQ animals had significantly lower ABFT (*P* = 0.05) and LFW (*P* = 0.06) than Qq? animals. Although we did not observe significant differences in ABFT (*P* = 0.15) and LFW (*P* = 0.17) between Qq and qq? animals, Qq individuals tended to have lower ABFT (2.95 ± 0.77 vs. 3.20 ± 0.41 cm) and LFW (2086.6 ± 1076.2 vs. 2416.3 ± 864. g) than qq? individuals. Altogether, our findings indicate that the q? haplotype is most likely a q haplotype that increases fat deposition, rather than a Q haplotype that decreases fat deposition. All the q-type haplotypes were distinct from the Q-type haplotypes in the 1.7-Mb Q-sharing segment (Figure 3C).

**Figure 4:**
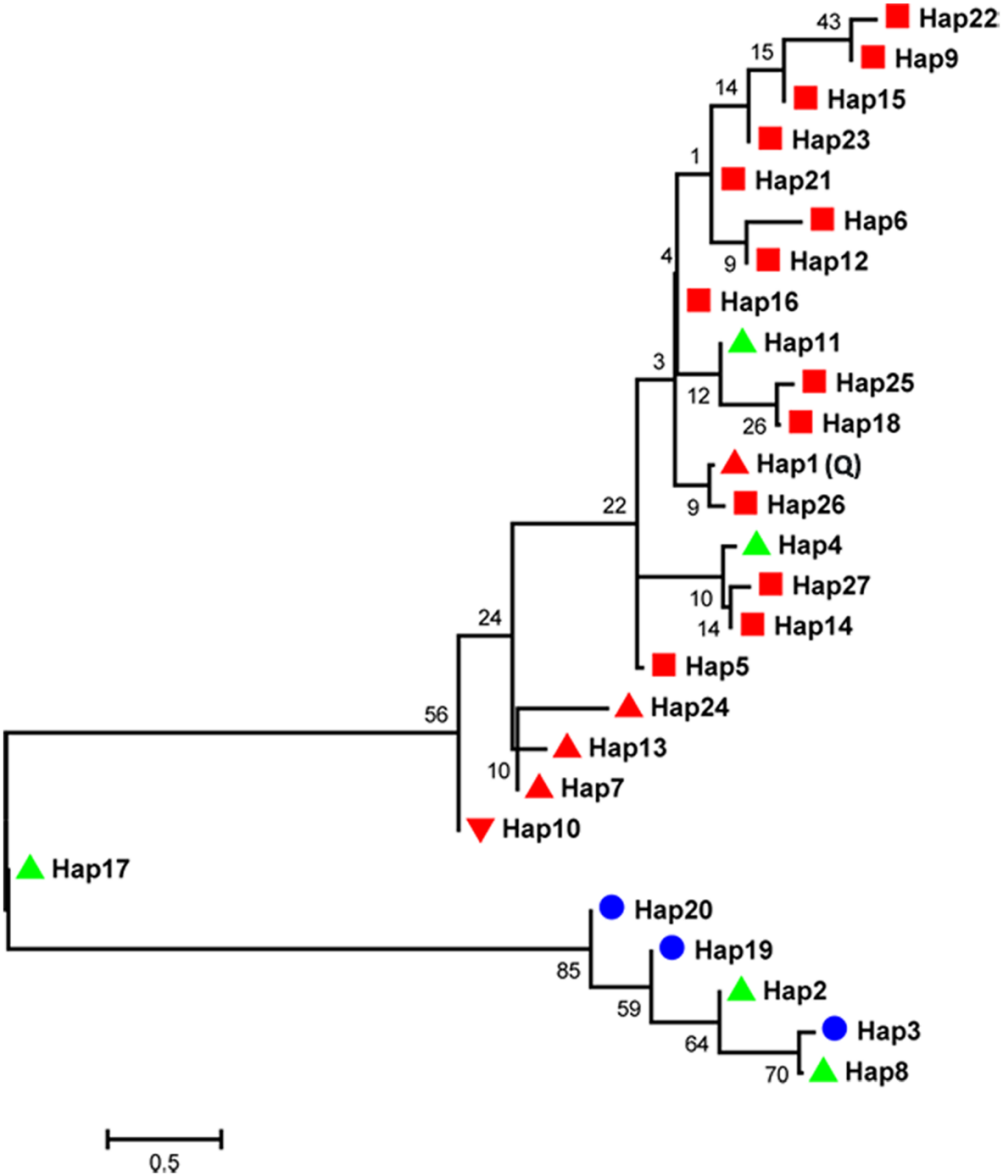
**Neighbor-joining tree of 27 major haplotypes for a 750-kb critical region of the major QTL on SSC7.** Haplotypes with frequencies greater than 0.05 were used to construct a NJ tree using the 750-kb critical region of SSC7. Hap 1 is the Q-bearing haplotype; haplotypes specific to Chinese indigenous breeds are indicated in red blocks; haplotypes present in both Chinese and Western breeds are highlighted in blue blocks; haplotypes prominently occurring in Chinese breeds and Western breeds are marked in red and green triangular blocks, respectively; haplotypes specific to Chinese indigenous breeds and Chinese synthetic breeds are indicated by red inverted triangular blocks.

We were particularly interested in a ~750-kb region on SSC7 (between 34 673 190 and 35 422 882 bp) that was contained within both the 2.1-Mb 95% CI and the 1.7-Mb Q-sharing segment (Figure 3C). It is most likely that this region encompasses the gene responsible for the SSC7 locus. To investigate the population genetics and evolutionary history at this SSC7 locus, we reconstructed haplotypes corresponding to the critical 750-kb region for 589 individuals from 31 diverse Chinese and Western breeds (Table 2), which had been genotyped for 62 163 SNPs using the Illumina Porcine 60 K DNA chip [25]. A total of 27 haplotypes with frequencies greater than 0.05 were subsequently used to construct a neighbor-joining (NJ) tree. The phylogenetic tree clearly illustrated that the Q-type haplotype is of Chinese origin (Figure 4). Of the 27 haplotypes, 14 occurred exclusively in Chinese indigenous pigs, and two were predominately (>0.8) present in Western pigs. Notably, the Q-type haplotype that we identified in the F_2_ population was present in multiple Chinese indigenous breeds, many of which carry the Q allele at considerably high frequencies (Table 2). Conversely, this haplotype was nearly absent in Western breeds and Chinese wild boars, which suggests that the causal variants underlying the SSC7 QTL originated after the domestication of Chinese wild boars. Further investigations in multiple Chinese breeds of the minimal shared haplotype that carries the Q allele would be useful to identify the causative mutation underlying the SSC7 locus. Given that a number of Chinese breeds segregate for this major locus, characterization of the causal variant would greatly contribute to the genetic improvement of growth and fatness traits in Chinese indigenous pigs.

**Table 2:** **Frequency of the Q-bearing haplotype at the SSC7 locus in 31 Chinese a** **nd Western pig breeds**

**Breed**	**Origin**	**Number**	**Frequency**
Indigenous Chinese breeds			
Erhualian	Jiangsu	32	0.83
Dongshan	Guangxi	15	0.73
Min	Heilongjiang	22	0.66
Hetao Large Ear	Inner Mongolia	16	0.59
Tongcheng	Hubei	16	0.59
Rongchang	Chongqing	18	0.58
Neijiang	Sichuan	16	0.56
Shaziling	Hunan	11	0.55
Kele	Guizhou	10	0.55
Guangdong Dahuabai	Guangdong	16	0.53
Luchuan	Guangxi	18	0.47
Ganxi	Jiangxi	13	0.46
Tibetan (Diqing)	Yunnan	19	0.34
Bamaxiang	Guangxi	16	0.34
Mingguang Small Ear	Yunnan	16	0.34
Bamei	Qinghai	16	0.31
Tibetan (Milin)	Tibet	16	0.25
Wuzhishan	Hainan	16	0.25
Diannan Small-ear	Yunnan	15	0.10
Congjiangxiang	Guizhou	16	0.09
Tibetan (Litang)	Sichuan	16	0.06
Tibetan (Hezuo)	Gansu	21	0.05
Tibetan (Gongbujiangda)	Tibet	29	0.03
Laiwu	Shandong	18	0.03
Wild boar	Jiangxi	20	0.00
Jinhua	Zhejiang	13	0.00
Licha Black	Shandong	14	0.00
Chinese synthetic breed			
Sutai	Jiangsu	15	0.03
Western commercial breeds			
Large White	France	35	0.01
Landrace	Denmark	35	0.00
Duroc	U.S.A	40	0.00

The 750-kb critical region on SSC7 harbors eight annotated genes, including *GRM4* (*glutamate receptor, metabotropic 4*), *HMGA1* (*high mobility group AT-hook 1*), *C6ORF1* (*chromosome 6 open reading frame 1*), *NUDT3* (*nudix (nucleoside diphosphate linked moiety X)-type motif 3*), *RSP10* (*ribosomal protein S10*), *SPDEF* (*SAM pointed domain containing ETS transcription factor*), *PACSIN1* (*protein kinase C and casein kinase substrate in neurons 1*) and *C6ORF106* (*chromosome 6 open reading frame 106*) (Figure 3C). Of these eight genes, *NUDT3* could be a candidate gene for the SSC7 locus, as *NUDT3* variants have been associated with changes in human body mass index values [33] and height [34]. However, *HMGA1* is another promising candidate gene since it is functionally related to growth and fat metabolism. It encodes a non-histone protein that plays a role in multiple cellular processes. HMGA1 can serve as a modulator of IGF1 (insulin-like growth factor 1) activity and consequently regulates glucose uptake [35]. It is also significantly associated with human height [36]. Moreover, a STRING protein-protein interaction network shows that HMGA1 can bind with PPARG (peroxisome proliferator-activated receptor gamma), a key regulator of fat-cell differentiation and glucose homeostasis [37] (see Additional file 7: Figure S4). In our previous study, we genotyped one *HMGA1* variant (g.3135C > T) in all individuals of the F_2_ population [32]. When we included this *HMGA1* SNP in the single-marker GWAS, it was the most significant SNP for backfat thickness on SSC7 (see Additional file 8: Figure S5) and exhibited the same strength of association as the top SNP of the original GWAS (ss107837325). This finding further supports *HMGA1* as a highly plausible candidate gene responsible for the SSC7 locus. It would thus be worthwhile to further investigate functional variants in *HMGA1* that underlie the QTL effect on SSC7.

The 750-kb interval is approximately 700 kb upstream of the *PPARD* gene (between 36 141 606 and 36 215 260 bp), which has been identified as the gene responsible for ear size in the F_2_ population [32]. Here, we show that *PPARD* falls outside the 95% CI of the SSC7 locus, which indicates that the growth and fatness traits associated with this locus are not affected by polymorphisms in the *PPARD* gene. When we included *PPARD* G32E SNP, the causal mutation for ear size, in the GWAS analysis of fatness traits, we found that this SNP had a much lower association significance than the top SNP ss107837325 (see Additional file 8: Figure S5). This finding provided more evidence that *PPARD* is not the gene responsible for the SSC7 locus that affects growth and fatness traits.

#### SSC4

The second strongest effect on the measured traits was observed within an interval of 7 Mb (between 82 and 89 Mb) on SSC4. After fitting the effect of the SSC7 locus, the strength of the association between this locus and fatness traits in F_2_ animals exceeded the genome-wide significance threshold (see Additional file 9: Figure S6). This region also showed various associations with growth traits in F_2_ animals, although the strength of the association only reached the chromosome-wide significance level (see Additional file 2: Table S2). The top SNP at this locus was ss131269801, at 82 850 635 bp. This locus corresponds to the well-known *FAT1* region, which was identified in the first genome scan for pig QTL [38] and subsequently characterized in multiple pig resource populations [4,39-41]. Multiple *FABP* (*fatty acid binding protein*) genes, such as *FABP4*, have been proposed as candidate genes for this locus [42,43]. However, the *FABP4* gene is located 9 Mb away from the top SNP in this study. When we corrected for the effect of the top SNP, the GWAS signal was entirely absent from SSC4. Thus, *FABP* genes are probably not the genes of interest here. Instead, just ~240 kb downstream from the top SNP lies the *PLAG1* (*pleiomorphic adenoma gene 1*) gene, which has been reported to be associated with bovine stature [44] and human height [36]. Moreover, *PLAG1* appears to be one of the top genes that was under selection during the domestication of European pigs, and *PLAG1* variants have been associated with growth and fatness traits in a European Wild boar × Large White F_2_ intercross population [45]. The *PLAG1* region harbors multiple SNPs that show strong signals of selection and marked differences in allele frequency between Asian pigs/wild boars and European domestic pigs [45]. To investigate this, we randomly chose one of the most strongly fixed SNPs in European domestic pigs at 82 499 373 bp on SSC4, and genotyped this *PLAG1* SNP by Sanger sequencing for all animals of the F_2_ population. The *PLAG1* SNP data were then incorporated into the 60 K SNP dataset for the single-marker GWAS, after correction for the effect of the SSC7 locus. Our results showed that the association between the *PLAG1* SNP and growth and fatness traits was as strong as that with the top SNP from the 60 K panel (ss131269801), which was attributable to complete linkage disequilibrium between the two SNPs (data not shown). Based on this, we propose that *PLAG1* is a strong candidate gene for the SSC4 locus. It should also be noted that another locus with significant effects on body weight at day 21 in Sutai pigs was identified in a different region on this chromosome, with the top SNP located at 134 935 929 bp.

#### SSC2

After correcting for the effects of the SSC7 locus, the effect of SSC2 on fatness traits surpassed the genome-wide significance threshold (see Additional file 9: Figure S6). The finding is consistent with our previous QTL mapping results [28]. The top SNP (ss131060419) was located at the distal end (920 370 bp) of SSC2, corresponding to the well-characterized *IGF2* gene, which affects muscle mass and fatness in pigs [46]. It is known that *IGF2* is a paternally expressed gene. However, the effect at the SSC2 locus disappeared when we applied a paternally-expressed-imprinting statistical model to the data (data not shown). Therefore, *IGF2* is likely not the gene responsible for this locus. Another region, around 101.69 Mb on SSC2 had a significant effect on body weight at day 21 in Sutai pigs.

#### SSCX

Our previous QTL scans and studies by other researchers have consistently found the presence of a major locus for fatness traits on SSCX based on data from Chinese × Western intercross populations [5,28,30,47-50]. Here, we confirm these previous findings by identifying a locus of genome-wide significance for backfat thickness at around 62 Mb on SSCX, with the top SNP (ss131561996) at 62 086 511 bp. This region has been shown to be a recombination cold spot that spans ~31 Mb and shows an extremely low rate of recombination in our F_2_ population [45,51]. This low-recombination region harbors a large number of predicted genes [28], which makes it impossible to identify plausible candidate genes.

### Loci of genome-wide significance and plausible candidate genes in Sutai pigs

A total of seven loci of genome-wide significance were identified in Sutai pigs, with one each on SSC2, 10 and 14, and two on SSC3 and two on SSC4 (Table 1). These loci were all associated with growth during the suckling period (from 0 to 21 days) in Sutai pigs but not in F_2_ pigs. The most significant (*P* = 1.22 × 10^−8^) locus was associated with a region on SSC3, but no obvious candidate gene was identified. A locus on SSC2, with its top SNP (ss107909052) at 101 694 375 bp (*P* = 2.66 × 10^−8^), corresponded to a QTL with chromosome-wide significance for body weight, which had been previously identified in a Chinese Meishan × Large White population [49]. *ARRDC3* (*arrestin domain containing 3*), ~125 kb away from this top SNP, appears to be a functionally related candidate gene at this locus. This gene has been reported to play a role in mechanisms that control the regulation of glucose and lipid metabolism and insulin secretion [52,53]. Moreover, the locus on SSC10 that had the strongest signal at 13 838 740 bp (*P* = 8.89 × 10^−7^), overlaps with a QTL of genome-wide significance that affects body weight at birth in Western hybrid pigs [54]. We did not find any apparent candidate gene in the region around the top SNP (ss478939281) at that locus. The QTL on SSC4 and SSC14 are, to our knowledge, detected for the first time in this study. There were no obvious candidate genes in the vicinity of the top SNPs (ss131247062, ss107817770 and ss131505908) at these two QTL.

### Suggestive QTL for growth and fatness traits

In addition to the significant loci mentioned above, we identified 39 QTL for which a chromosome-wide, but not a genome-wide, association was detected with 20 growth and fatness traits (see Additional file 2: Table S2). These QTL included two each on SSC1, 3, 4, 9 and 15; seven on SSC2; three each on SSC5, 6, 7, and 8; one each on SSC10, 11, 12, 18 and X; and five on SSC14. However, most of these QTL were not shared between the F_2_ and Sutai populations. The suggestive QTL on SSC2, 4 and 7 overlapped perfectly with the above-described prominent QTL in the F_2_ population, since identical top SNPs were observed at these loci. The multiple associations clearly indicate a pleiotropic role of these QTL in regulation of growth and fat deposition in pigs. In addition, SSC14 showed time-dependent effects on body weight at three distinct regions. Each region was associated with changes in body weight at a different time point (days 0, 21 or 240) in Sutai pigs.

## Discussion

### GWAS versus traditional QTL mapping

Compared to traditional QTL mapping approaches, one obvious advantage of GWAS is that it can use high-density markers along the entire genome and thus capture enough LD (linkage disequilibrium) to identify a majority of potential causal variants. The GWAS top SNPs are usually in the vicinity of causal mutations, allowing us to more accurately pinpoint the most likely candidate genes for the loci of interest. In F_2_ crosses, clusters of significant SNPs are usually revealed at the major QTL by long-range LD patterns. Further haplotype-sharing analyses based on these significant SNPs can then be used to refine the location of the QTL. By using GWAS and haplotype-sharing analysis, we identified *HMGA1* and *PLAG1* as promising candidate genes at the prominent loci on SSC7 and SSC4, respectively. This finding has the potential to significantly benefit the ultimate characterization of the underlying variation in these loci in the near future.

We note that fewer significant loci were detected by GWAS in this study than in a previous study in which we used QTL mapping in the same White Duroc × Erhualian F_2_ intercross population [28]. This inconsistency could be caused by two factors. First, GWAS employs a more stringent Bonferroni-corrected threshold. This conservative threshold reduces the false discovery rate but simultaneously decreases the ability to detect loci with moderate or small effects. Second, only additive SNP effects were included in the mixed linear GWAS model while dominant effects were also considered in the QTL model.

### Shared and unique loci in the F_2_ and Sutai populations

In this study, we did not observe any locus of genome-wide significance that was shared between the White Duroc × Erhualian F_2_ intercross and Sutai populations, a finding that highlights the complex genetic architecture of growth and fatness traits. Sutai pigs were originally developed from a cross between Chinese Taihu pigs (including Erhualian and Meishan pigs) and Duroc pigs and have been artificially selected for at least 18 generations for lean meat and litter size [2]. In theory, some GWAS signals should be shared between the White Duroc × Erhualian F_2_ intercross pigs and the Sutai pigs due to their similar genomic backgrounds. In fact, we have detected both shared and distinct GWAS results for multiple phenotypic traits in the two populations [55-57]. To test why the prominent loci associated with fatness and growth traits on SSC2, 4, 7 and X that were identified in the intercross pigs are absent from Sutai pigs, we further examined the most significant locus on SSC7. We found that the Q-bearing haplotype that corresponded to the critical region of this locus was not inherited by the current Sutai population from its Chinese Taihu founders; indeed, in the current Sutai population, all individuals are homozygous for the q-type haplotype. This fact partly explains why the SSC7 QTL effect has disappeared in Sutai pigs. However, we are unable to rule out other possibilities for certain. The molecular basis that underlies porcine growth and fatness traits could be more complicated than expected.

## Conclusions

We performed a GWAS of 14 growth and eight fatness traits in a White Duroc × Erhualian F_2_ intercross population and a Chinese Sutai half-sib population. Fourteen QTL of genome-wide significance and 39 suggestive loci of chromosome-wide significance were identified on 16 chromosomes. The discovery of strongly associated loci on SSC2, 4, 7 and X confirms our previous QTL mapping results. The critical region of the locus on SSC7 was refined to a ~750 kb segment, and the Q allele that promotes fast growth and decreases fat deposition was confirmed to be of Chinese origin and probably arose after the domestication of Chinese wild boars. Population genetic analysis revealed that geographically diverse Chinese breeds segregate for the Q allele, which illustrates the importance of this allele in genetic improvement of Chinese indigenous pigs. Several promising candidate genes were identified, including *HMGA1* at the SSC7 locus and *PLAG1* at the SSC4 locus. No significant loci were shared between the Sutai and F_2_ populations, and both time-constant and time-specific loci were detected in association with growth traits at different stages, which illustrates the complexity of the molecular mechanisms that underlie growth and fatness in pigs. Our findings provide novel insights into the genetic basis of growth and fatness in pigs and may contribute to the identification of the causal variants for the identified loci, especially for the major loci on SSC4 and SSC7.

## Additional files

Additional file 1: Table S1. Descriptive statistics for growth and fatness traits in the tested samples. This table provides data on the descriptive statistics for growth and fatness traits in the tested samples. Additional file 2: Table S2. Suggestive loci identified by GWAS for growth and fatness traits in White Duroc × Erhualian F_2_ pigs and Sutai pigs. The table presents the loci of chromosome-wide significance that were identified by GWAS for growth and fatness traits in White Duroc × Erhualian F_2_ pigs and Sutai pigs. Additional file 3: Figure S1. Manhattan plots for the analyses of fatness traits in Sutai pigs. The GWAS for ABFT **(A)**, LFW **(B)**, VFW **(C)** and AFW **(D)** in Sutai pigs. In the Manhattan plots, negative log_10_ *P* values of the qualified SNPs were plotted against their genomic positions. The SNPs on different chromosomes are denoted by different colors. The solid and dashed lines indicate the 5% genome-wide and chromosome-wide (i.e., suggestive) Bonferroni-corrected thresholds, respectively. ABFT: average backfat thickness; LFW: leaf fat weight; VFW: veil fat weight; AFW: abdominal fat weight. Additional file 4: Figure S2. Box-and-whisker plot for the effect of haplotypes corresponding to the 1.7-Mb critical region of the SSC7 locus on fat deposition in F_2_ animals. Phenotypic values of average backfat thickness (ABFT) and leaf fat weight (LFW) are on the Y-axis. N indicates the number of each haplotype. Haplotype 1, from 13 Erhualian founder sows, is associated with decreased fat deposition and is thus defined as the Q-bearing haplotype. Haplotypes 2, 3, 4 and 5 are associated with increased fat deposition and thus considered to be the q-bearing haplotype. Haplotypes 2 and 3 were inherited from two White Duroc founder sires (73 and 75) and haplotype 4 from two Erhualian F_0_ sows (124 and 126). Haplotype 5 is a recombinant haplotype between haplotypes 2 and 3. Haplotypes 2, 3 and 4 correspond to the q1, q2 and q3 chromosomes in Figure 3B, respectively. Haplotype 6, which is indicated by “?”, was inherited from Erhualian F_0_ sows 142 and 146. Of the 930 F_2_ animals genotyped for ~62 000 SNPs using the Illumina porcine 60 K DNA chip, only 10 F_2_ individuals carried this haplotype, and its QTL status could not be deduced (Panel **A**). Panel **B** shows the effect of this haplotype on average backfat thickness and leaf fat weight after the inclusion of seven additional individuals carrying the haplotype. Additional file 5: Figure S3. Pedigree of 17 F_2_ and F_3_ individuals carrying the q? haplotype. All 17 individuals are indicated in green. Note that individuals 1881 and 2086 had not been recorded for fatness traits at slaughter since these individuals were used to produce F_3_ offspring. Additional file 6: Table S3. Phenotypic differences in average backfat thickness and leaf fat weight among the Q, q and q? haplotypes. This table provides data that illustrate the phenotypic differences in average backfat thickness and leaf fat weight among the Q, q and q? haplotypes. Additional file 7: Figure S4. Known and predicted protein-protein interactions of HMGA1 in the STRING database (available at http://string-db.org). HMGA1 can bind with PPARG (peroxisome proliferator-activated receptor gamma), a key regulator of fat-cell differentiation and glucose homeostasis. Additional file 8: Figure S5. Manhattan plots for the analyses of backfat thickness at the first rib in F_2_ animals using the 60 K-chip SNPs, *PPARD* G32E and *HMGA1* g.3135C > T on SSC7. In the Manhattan plots, negative log_10_ *P* values of the filtered high-quality SNPs were plotted against their genomic positions. *PPARD* G32E and *HMGA1* g.3135C > T SNPs are highlighted in green and red, respectively. Additional file 9: Figure S6. Manhattan plots for the analyses of fatness traits after accounting for the effect of the SSC7 locus in F_2_ animals. Description: In the Manhattan plots, negative log_10_ *P* values of the filtered high-quality SNPs were plotted against their genomic positions after correcting for the effect of *HMGA1* g.3135C > T. The SNPs on different chromosomes are denoted by different colors. The solid and dashed lines indicate the 5% genome-wide and chromosome-wide (i.e., suggestive) Bonferroni-corrected thresholds, respectively. ABFT: average backfat thickness; LFW: leaf fat weight; VFW: veil fat weight; AFW: abdominal fat weight.
