# Supplementary material for: Genome-wide association analyses reveal significant loci and strong candidate genes for growth and fatness traits in two pig populations
Source: Genet Sel Evol. 2015 Mar 14;47(1):17. doi: 10.1186/s12711-015-0089-5 (PMC4358731; doi:10.1186/s12711-015-0089-5)
Supplement: Additional file 1: Table S1. — Descriptive statistics for growth and fatness traits in the tested samples. This table provides data on the descriptive statistics for growth and fatness traits in the tested samples. [file 12711_2015_89_MOESM1_ESM.doc]

**Table S1 Descriptive statistics for growth and fatness traits in the tested samples.**

| Trait | Symbol | F_2_ pigs |  |  |  | Sutai pigs |  |  |  |
| --- | --- | --- | --- | --- | --- | --- | --- | --- | --- |
|  |  | No. | Mean ± SD | Min | Max | No. | Mean ± SD | Min | Max |
| Growth |  |  |  |  |  |  |  |  |  |
| Birth weight, kg | BW0 | 927 | 1.22 ± 0.24 | 0.55 | 2.05 | 425 | 1.13 ± 0.25 | 0.54 | 2.07 |
| Body weight at 21 day, kg | BW21 | 925 | 5.35 ± 1.21 | 1.4 | 9.2 | 184 | 4.43 ± 0.75 | 2.84 | 6.94 |
| Body weight at 46 day, kg | BW46 | 928 | 11.40 ± 2.64 | 3 | 20.1 | - | - | - | - |
| Body weight at 120 day, kg | BW120 | 291 | 31.71 ± 6.42 | 13 | 53.5 | 432 | 31.63 ± 7.14 | 7.8 | 54 |
| Body weight at 210 day, kg | BW210 | 743 | 83.71 ± 15.10 | 31 | 132 | 419 | 66.04 ± 15.50 | 15.5 | 107.5 |
| Body weight at 240 day, kg | BW240 | 929 | 97.27 ± 17.88 | 26.6 | 146.2 | 408 | 75.58 ± 16.33 | 18 | 122 |
| Average daily gain from 0 to 21 day, kg | ADG0-21 | - | - | - | - | 184 | 0.16 ± 0.03 | 0.1 | 0.25 |
| Average daily gain from 0 to 46 day, kg | ADG0-46 | 925 | 0.22 ± 0.06 | 0.05 | 0.39 | - | - | - | - |
| Average daily gain from 21 to 120 day, kg | ADG21-120 | - | - | - | - | 184 | 0.26 ± 0.06 | 0.11 | 0.44 |
| Average daily gain from 46 to 120 day, kg | ADG46-120 | 291 | 0.27 ± 0.07 | 0.04 | 0.56 | - | - | - | - |
| Average daily gain from 120 to 240 day, kg | ADG120-240 | 290 | 0.54 ± 0.12 | 0.23 | 0.87 | 408 | 0.36 ± 0.10 | 0.05 | 0.65 |
| Average daily gain from 21 to 240 day, kg | ADG21-240 | - | - | - | - | 177 | 0.31 ± 0.06 | 0.16 | 0.46 |
| Average daily gain from 46 to 240 day, kg | ADG46-240 | 927 | 0.45 ± 0.09 | 0.18 | 0.68 | - | - | - | - |
| Average daily gain from 0 to 240 day, kg | ADG0-240 | - | - | - | - | 399 | 0.31 ± 0.07 | 0.07 | 0.51 |
| Fatness |  |  |  |  |  |  |  |  |  |
| Backfat thickness at the shoulder, cm | SBFT | 930 | 3.96 ± 0.95 | 1.34 | 7.3 | 408 | 2.95 ± 0.86 | 0.49 | 5.89 |
| Backfat thickness at the first rib, cm | FRBFT | 930 | 3.16 ± 0.97 | 0.14 | 6.85 | 408 | 2.43 ± 0.80 | 0.15 | 5.01 |
| Backfat thickness at the last rib, cm | LRBFT | 930 | 2.37 ± 0.87 | 0.21 | 6.93 | 408 | 1.59 ± 0.60 | 0.2 | 3.13 |
| Backfat thickness at the hip, cm | HBFT | 930 | 2.57 ± 1.02 | 0.24 | 7.01 | 408 | 1.74 ± 0.80 | 0.15 | 4.27 |
| Average backfat thickness, cm | ABFT | 930 | 3.02 ± 0.88 | 0.48 | 6.51 | 408 | 2.18 ± 0.72 | 0.27 | 4.38 |
| Leaf fat weight, kg | LFW | 926 | 2.12 ± 1.11 | 0.07 | 6.06 | 408 | 1.22 ± 0.64 | 0.05 | 3.31 |
| Veil fat weight, kg | VFW | 928 | 1.32 ± 0.45 | 0.22 | 3.35 | 403 | 0.24 ± 0.10 | 0.01 | 0.59 |
| Abdominal fat weight, kg | AFW | 928 | 1.24 ± 0.43 | 0.11 | 2.74 | 408 | 0.69 ± 0.30 | 0.04 | 1.9 |
