# Supplementary material for: Genome-wide association analyses reveal significant loci and strong candidate genes for growth and fatness traits in two pig populations
Source: Genet Sel Evol. 2015 Mar 14;47(1):17. doi: 10.1186/s12711-015-0089-5 (PMC4358731; doi:10.1186/s12711-015-0089-5)
Supplement: Additional file 2: Table S2. — Suggestive loci identified by GWAS for growth and fatness traits in White Duroc × Erhualian F2 pigs and Sutai pigs. The table presents the loci of chromosome-wide significance that were identified by GWAS for growth and fatness traits in White Duroc × Erhualian F2 pigs and Sutai pigs. [file 12711_2015_89_MOESM2_ESM.doc]

**Table S2** Suggestive loci identified by GWAS for growth and fatness traits in White Duroc × Erhualian F_2_ animals and Sutai pigs.

| Chr ^1^ | Trait | Population | Top SNP | Position (bp) | MAF ^2^ | *P* value | Consistence with QTL^3^ |
| --- | --- | --- | --- | --- | --- | --- | --- |
| 1 | LFW | Sutai | ss131179316 | 12459957 | 0.18 | 8.23E-06 | N |
|  | VFW | Sutai | ss131179316 | 12459957 | 0.18 | 4.65E-06 | N |
|  | ADG0-120 | Sutai | ss131179316 | 12459957 | 0.19 | 2.42E-05 | - |
|  | FRBFT | Sutai | ss131139967 | 24034707 | 0.12 | 1.93E-05 | N |
| 2 | ABFT | F_2_ | ss131122224 | 1249101 | 0.28 | 2.61E-06 | Y |
|  | FRBFT | F_2_ | ss131122224 | 1249101 | 0.28 | 1.36E-05 | Y |
|  | AFW | F_2_ | ss107908103 | 963130 | 0.27 | 7.29E-06 | Y |
|  | LFW | F_2_ | ss107879231 | 1024375 | 0.33 | 2.10E-05 | Y |
|  | FRBFT | Sutai | ss131196667 | 10286572 | 0.40 | 8.26E-06 | Y |
|  | BW46 | F_2_ | ss131036858 | 148611135 | 0.21 | 2.63E-05 |  |
|  | ADG0-46 | F_2_ | ss131036858 | 148611135 | 0.21 | 2.63E-05 | - |
|  | ADG0-21 | Sutai | ss107909052 | 101694375 | 0.05 | 5.00E-06 | - |
|  | ADG21-120 | Sutai | ss107859939 | 18665152 | 0.08 | 1.68E-05 | - |
| 3 | BW0 | Sutai | ss131230651 | 127121184 | 0.29 | 9.10E-06 | N |
|  | VFW | Sutai | ss131226417 | 15450089 | 0.21 | 2.05E-05 |  |
| 4 | LRBFT | F_2_ | ss131269801 | 82850635 | 0.46 | 6.32E-06 | N |
|  | AFW | F_2_ | ss131269801 | 82850635 | 0.46 | 1.30E-05 | N |
|  | ADG46-240 | F_2_ | ss478941017 | 83025851 | 0.48 | 2.47E-05 | - |
|  | BW240 | F_2_ | ss478941017 | 83025851 | 0.48 | 1.88E-05 | N |
| 5 | BW240 | F_2_ | ss131094004 | 84399219 | 0.48 | 6.65E-06 | Y |
|  | ADG46-240 | F_2_ | ss131094004 | 84399219 | 0.48 | 1.29E-05 | - |
|  | LRBFT | F_2_ | ss131283599 | 32753183 | 0.46 | 8.73E-06 | N |
|  | BW21 | F_2_ | ss131072460 | 423263 | 0.22 | 2.03E-05 | N |
| 6 | BW21 | Sutai | ss131125917 | 139538709 | 0.05 | 6.92E-06 | N |
|  | ADG0-21 | Sutai | ss131030370 | 145742078 | 0.17 | 1.30E-06 | - |
|  | BW120 | Sutai | ss107862749 | 1619258 | 0.08 | 1.84E-05 | N |
|  | ADG0-120 | Sutai | ss107862749 | 1619258 | 0.08 | 2.11E-05 | - |
| 7 | BW240 | F_2_ | ss107837325 | 34803564 | 0.46 | 8.92E-06 | Y |
|  | ADG46-240 | F_2_ | ss107837325 | 34803564 | 0.46 | 1.83E-06 | - |
|  | ADG120-240 | F_2_ | ss131342496 | 32957768 | 0.49 | 2.44E-06 | - |
|  | ADG46-120 | F_2_ | ss131324493 | 113357835 | 0.06 | 2.40E-06 | - |
| 8 | VFW | F_2_ | ss131369469 | 6295586 | 0.14 | 7.20E-06 | N |
|  | SBFT | Sutai | ss131371720 | 78870676 | 0.08 | 7.42E-06 | Y |
|  | ADG0-21 | Sutai | ss131117063 | 146796310 | 0.14 | 2.16E-05 | - |
| 9 | BW0 | Sutai | ss131397048 | 70797373 | 0.05 | 3.20E-06 | N |
|  | VFW | Sutai | ss131410085 | 145153256 | 0.23 | 6.56E-06 | N |
| 10 | BW21 | Sutai | ss478939281 | 13838740 | 0.08 | 1.20E-05 | N |
| 11 | AFW | Sutai | ss131443342 | 48894442 | 0.06 | 1.65E-05 | N |
| 12 | BW21 | F_2_ | ss131472094 | 8626227 | 0.28 | 9.32E-06 | N |
| 14 | BW0 | Sutai | ss107897804 | 28225804 | 0.26 | 6.57E-06 | N |
|  | BW21 | Sutai | ss131505908 | 147757181 | 0.09 | 2.04E-06 | N |
|  | BW240 | Sutai | ss107896991 | 63223118 | 0.08 | 2.49E-05 | Y |
|  | FRBFT | Sutai | ss131506631 | 149969284 | 0.08 | 9.18E-06 | N |
|  | BW210 | F_2_ | ss131498350 | 113554994 | 0.37 | 2.49E-05 | N |
| 15 | ADG0-21 | Sutai | ss131528623 | 130599165 | 0.09 | 1.49E-05 | - |
|  | BW21 | F_2_ | ss107796512 | 143987209 | 0.12 | 2.46E-05 | N |
| 18 | BW21 | F_2_ | ss131557417 | 48660004 | 0.15 | 1.70E-05 | N |
| X | BW21 | Sutai | ss478944012 | 64665979 | 0.30 | 1.30E-05 | N |

^1^ Chromosome

^2^ Minor allele frequency

^3^ Replicated or novel loci compared to our previously identified QTL in the F_2_ cross [16].
