# Supplementary figures and images for: Genome-wide association analyses reveal significant loci and strong candidate genes for growth and fatness traits in two pig populations
Source: Genet Sel Evol. 2015 Mar 14;47(1):17. doi: 10.1186/s12711-015-0089-5 (PMC4358731; doi:10.1186/s12711-015-0089-5)

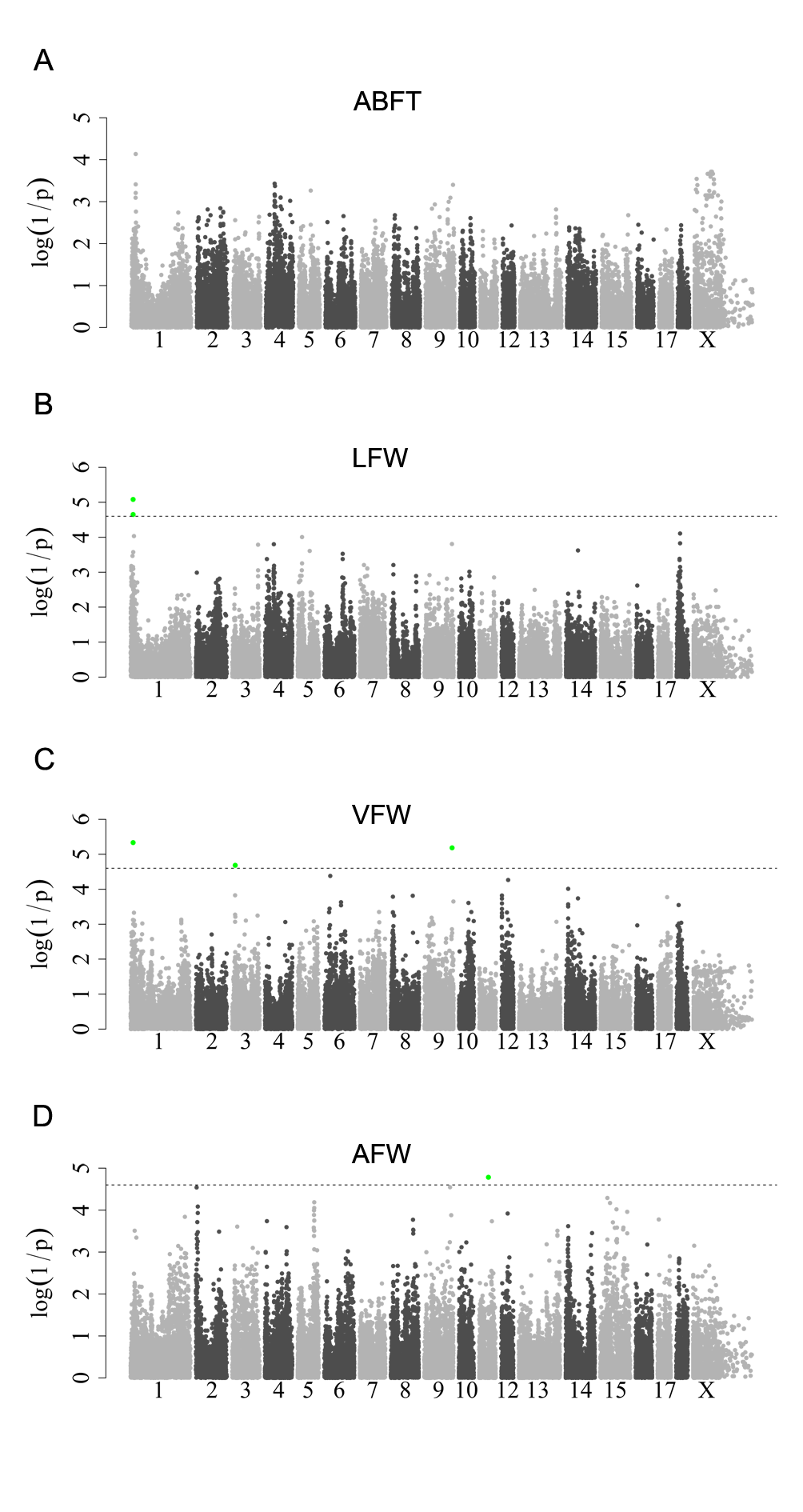

Supplement: Additional file 3: Figure S1. — Manhattan plots for the analyses of fatness traits in Sutai pigs. The GWAS for ABFT (A), LFW (B), VFW (C) and AFW (D) in Sutai pigs. In the Manhattan plots, negative log10 P values of the qualified SNPs were plotted against their genomic positions. The SNPs on different chromosomes are denoted by different colors. The solid and dashed lines indicate the 5% genome-wide and chromosome-wide (i.e., suggestive) Bonferroni-corrected thresholds, respectively. ABFT: average backfat thickness; LFW: leaf fat weight; VFW: veil fat weight; AFW: abdominal fat weight. [file 12711_2015_89_MOESM3_ESM.tiff]

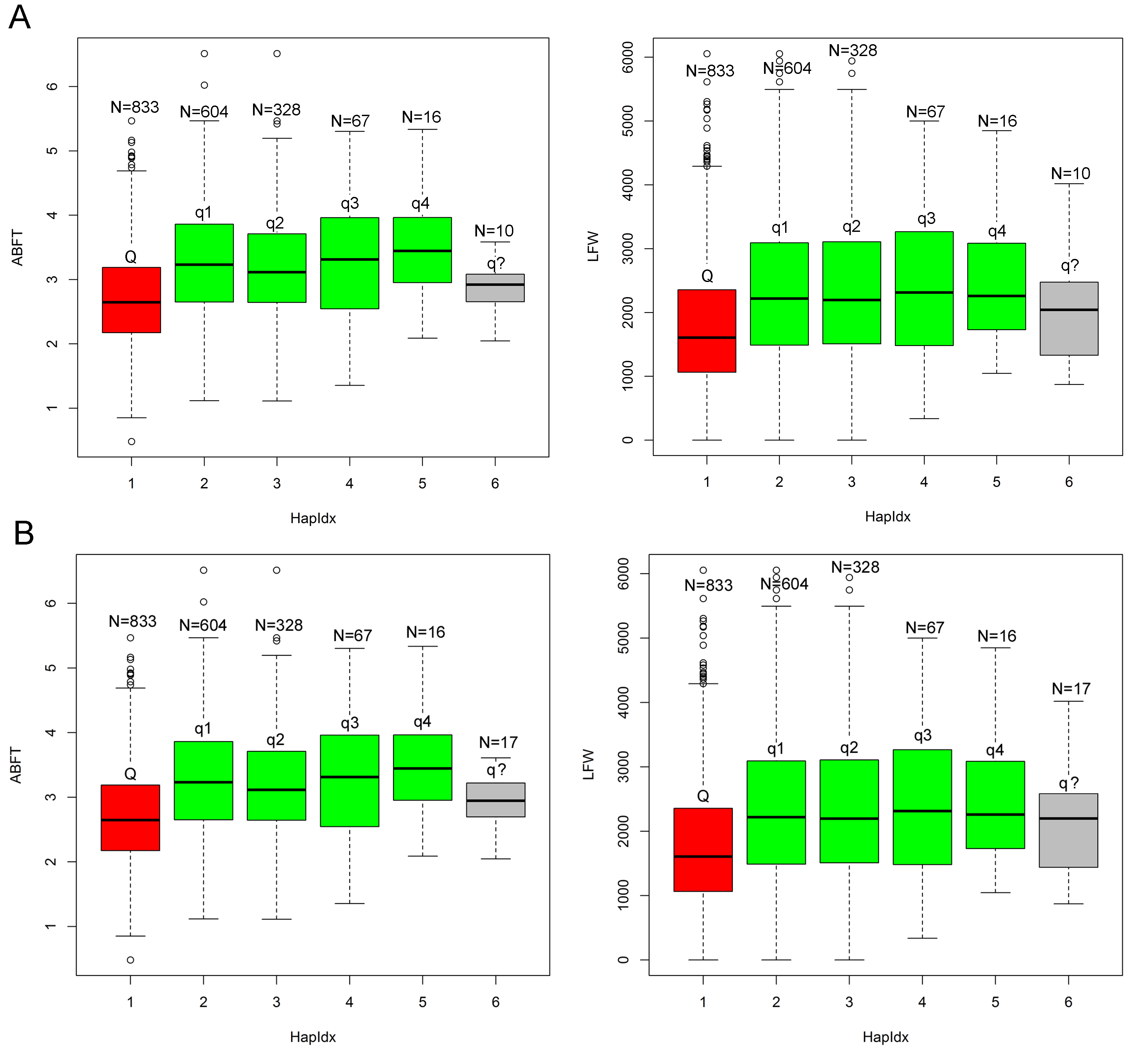

Supplement: Additional file 4: Figure S2. — Box-and-whisker plot for the effect of haplotypes corresponding to the 1.7-Mb critical region of the SSC7 locus on fat deposition in F2 animals. Phenotypic values of average backfat thickness (ABFT) and leaf fat weight (LFW) are on the Y-axis. N indicates the number of each haplotype. Haplotype 1, from 13 Erhualian founder sows, is associated with decreased fat deposition and is thus defined as the Q-bearing haplotype. Haplotypes 2, 3, 4 and 5 are associated with increased fat deposition and thus considered to be the q-bearing haplotype. Haplotypes 2 and 3 were inherited from two White Duroc founder sires (73 and 75) and haplotype 4 from two Erhualian F0 sows (124 and 126). Haplotype 5 is a recombinant haplotype between haplotypes 2 and 3. Haplotypes 2, 3 and 4 correspond to the q1, q2 and q3 chromosomes in Figure 3B, respectively. Haplotype 6, which is indicated by “?”, was inherited from Erhualian F0 sows 142 and 146. Of the 930 F2 animals genotyped for ~62 000 SNPs using the Illumina porcine 60 K DNA chip, only 10 F2 individuals carried this haplotype, and its QTL status could not be deduced (Panel A). Panel B shows the effect of this haplotype on average backfat thickness and leaf fat weight after the inclusion of seven additional individuals carrying the haplotype. [file 12711_2015_89_MOESM4_ESM.tiff]

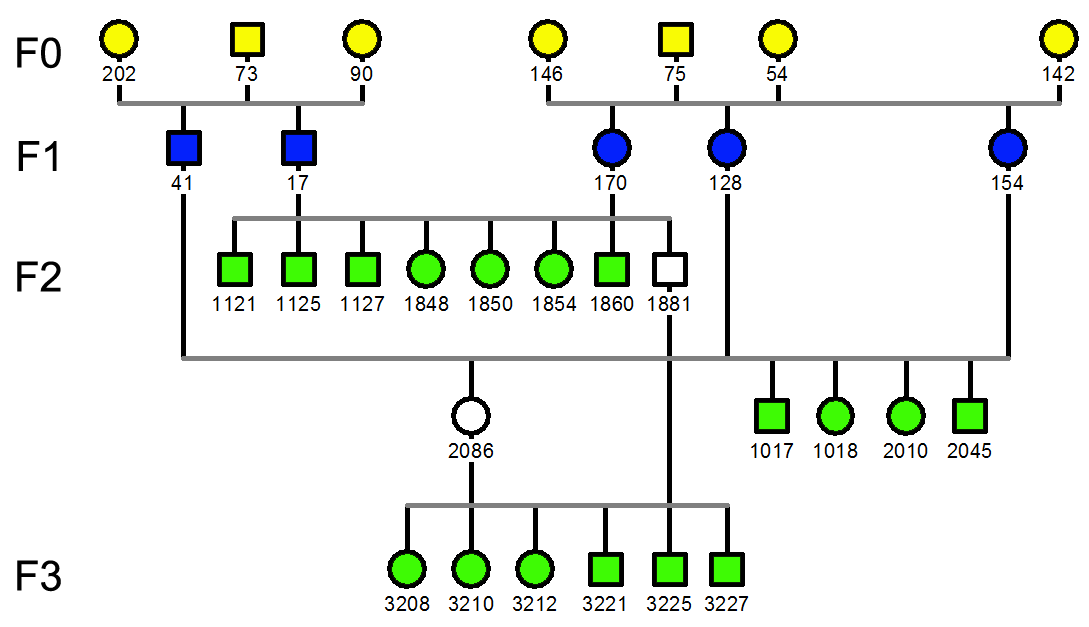

Supplement: Additional file 5: Figure S3. — Pedigree of 17 F2 and F3 individuals carrying the q? haplotype. All 17 individuals are indicated in green. Note that individuals 1881 and 2086 had not been recorded for fatness traits at slaughter since these individuals were used to produce F3 offspring. [file 12711_2015_89_MOESM5_ESM.tiff]

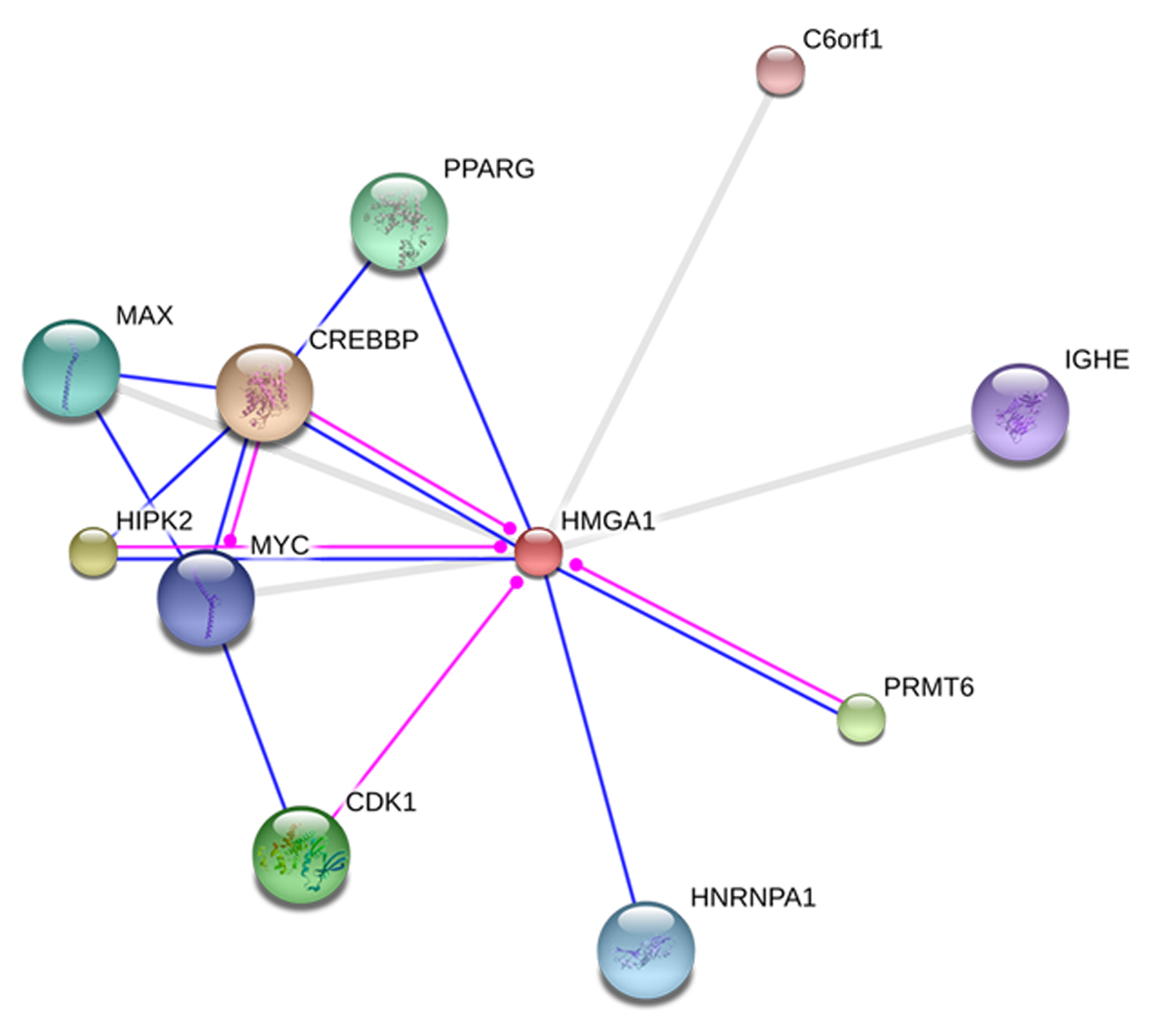

Supplement: Additional file 7: Figure S4. — Known and predicted protein-protein interactions of HMGA1 in the STRING database (available at http://string-db.org). HMGA1 can bind with PPARG (peroxisome proliferator-activated receptor gamma), a key regulator of fat-cell differentiation and glucose homeostasis. [file 12711_2015_89_MOESM7_ESM.tiff]

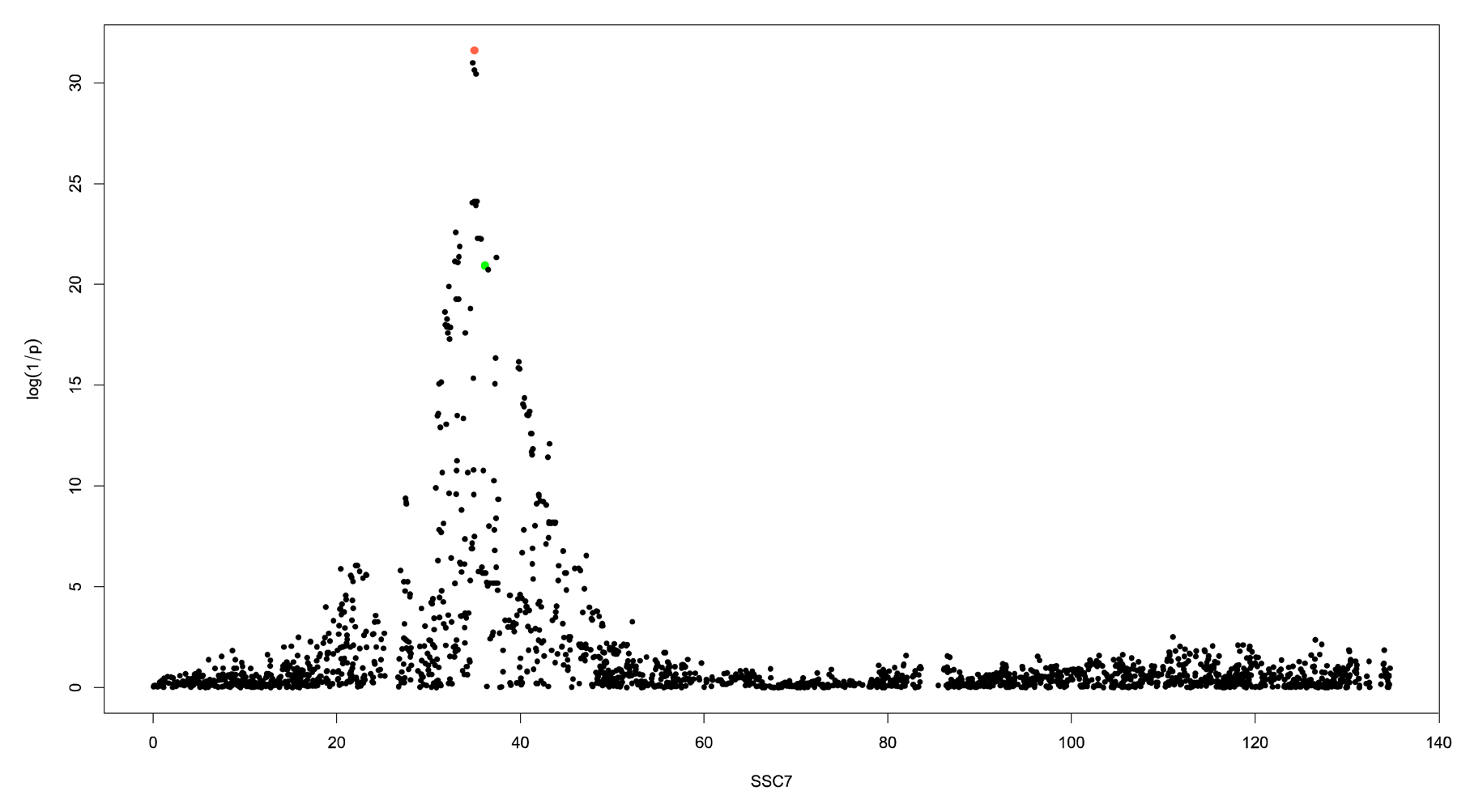

Supplement: Additional file 8: Figure S5. — Manhattan plots for the analyses of backfat thickness at the first rib in F2 animals using the 60 K-chip SNPs, PPARD G32E and HMGA1 g.3135C > T on SSC7. In the Manhattan plots, negative log10 P values of the filtered high-quality SNPs were plotted against their genomic positions. PPARD G32E and HMGA1 g.3135C > T SNPs are highlighted in green and red, respectively. [file 12711_2015_89_MOESM8_ESM.tiff]

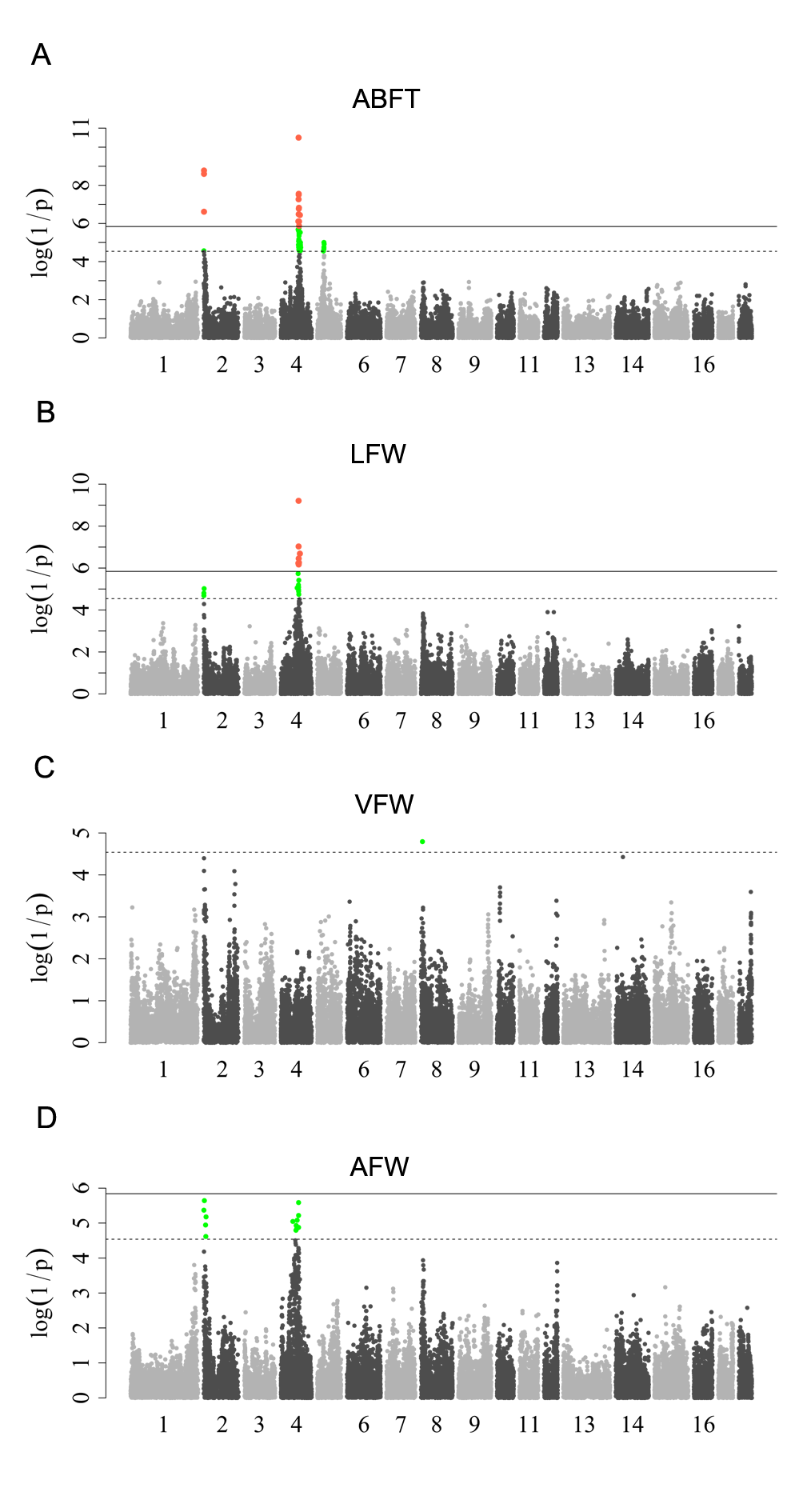

Supplement: Additional file 9: Figure S6. — Manhattan plots for the analyses of fatness traits after accounting for the effect of the SSC7 locus in F2 animals. Description: In the Manhattan plots, negative log10 P values of the filtered high-quality SNPs were plotted against their genomic positions after correcting for the effect of HMGA1 g.3135C > T. The SNPs on different chromosomes are denoted by different colors. The solid and dashed lines indicate the 5% genome-wide and chromosome-wide (i.e., suggestive) Bonferroni-corrected thresholds, respectively. ABFT: average backfat thickness; LFW: leaf fat weight; VFW: veil fat weight; AFW: abdominal fat weight. [file 12711_2015_89_MOESM9_ESM.tiff]
