# Supplementary material for: Genome-wide association analyses reveal significant loci and strong candidate genes for growth and fatness traits in two pig populations
Source: Genet Sel Evol. 2015 Mar 14;47(1):17. doi: 10.1186/s12711-015-0089-5 (PMC4358731; doi:10.1186/s12711-015-0089-5)
Supplement: Additional file 6: Table S3. — Phenotypic differences in average backfat thickness and leaf fat weight among the Q, q and q? haplotypes. This table provides data that illustrate the phenotypic differences in average backfat thickness and leaf fat weight among the Q, q and q? haplotypes. [file 12711_2015_89_MOESM6_ESM.doc]

**Table S3** Phenotypic difference in average backfat thickness and leaf fat weight among Q, q and q? haplotypes ^a^

| Trait | Phenotypic value |  |  |  |  | *P*-value |  |  |  |
| --- | --- | --- | --- | --- | --- | --- | --- | --- | --- |
|  | QQ  (n = 176) | qq  (n = 268) | Qq  (n = 474) | qq?  (n = 10) | Qq?  (n = 7) | qq - qq? | Qq - Qq? | Qq - qq? | QQ - Qq? |
| ABFT (cm) | 2.37 ± 0.67 | 3.56 ± 0.87 | 2.95 ±0 .77 | 3.20 ± 0.41 | 2.79 ± 0.41 | 0.10 | 0.29 | 0.15 | 0.05 |
| LFW (g) | 1403.46 ± 772.82 | 2630.29 ± 1117.14 | 2086.60 ± 1076.18 | 2416.30 ± 864.05 | 1863.57 ± 574.58 | 0.28 | 0.29 | 0.17 | 0.06 |

^a^ ABFT, average backfat thickness; LFW, leaf fat weight.. The q-type haplotype includes haplotypes q1, q2, q3 and q4 as shown in Figure S2. The pair-wise *t*-test was performed using the R software package.
